# Treatment of Odontogenic Maxillary Sinusitis with the Use of Growth Factors in Advanced Platelet-Rich Fibrin for Immediate Closure of Oro-Antral Communication: A Case Report

**DOI:** 10.3390/ijms25084339

**Published:** 2024-04-14

**Authors:** Paulina Adamska, Dorota Pylińska-Dąbrowska, Marcin Stasiak, Magdalena Kaczoruk-Wieremczuk, Ewa Kozłowska, Adam Zedler, Michał Studniarek

**Affiliations:** 1 Division of Oral Surgery, Faculty of Medicine, Medical University of Gdańsk, 7 Dębinki Street, 80-210 Gdańsk, Poland; adam.zedler@gumed.edu.pl; 2 Department of Dental Prosthetics, Faculty of Medicine, Medical University of Gdańsk, 18 Orzeszkowej Street, 80-204 Gdańsk, Poland; dorota.pylinska-dabrowska@gumed.edu.pl; 3 Division of Orthodontics, Faculty of Medicine, Medical University of Gdańsk, Aleja Zwycięstwa 42c, 80-210 Gdańsk, Poland; marcin.stasiak@gumed.edu.pl; 4 Individual Specialist Oral Surgery Practice Magdalena Kaczoruk-Wieremczuk, 41/31 Władysława Wysockiego Street, 17-100 Bielsk Podlaski, Poland; magdalena_kaczoruk@o2.pl; 5 Institute of Manufacturing and Materials Technology, Faculty of Mechanical Engineering and Ship Technology, Gdańsk University of Technology, 11/12 Gabriela Narutowicza Street, 80-233 Gdańsk, Poland; ewa.kozlowska@pg.edu.pl; 6 Department of Radiology, Faculty of Medicine, Medical University of Gdańsk, 17 Smoluchowskiego Street, 80-210 Gdańsk, Poland; michal.studniarek@gumed.edu.pl

**Keywords:** maxillary sinusitis, growth factor, platelet-rich fibrin, plasma, bone regeneration, autografts, oro-antral fistula

## Abstract

Chronic odontogenic maxillary sinusitis (COMS), a prolonged inflammation of the maxillary sinus lasting over 12 weeks, is often a result of periapical lesions, marginal periodontitis, and complications like oro-antral communication (OAC) and fistula (OAF). OAC, commonly emerging post-teeth extraction in the lateral maxilla, lacks documented treatments using advanced platelet-rich fibrin (A-PRF). This study evaluates A-PRF’s efficacy in treating COMS and immediately sealing extensive OAC. A case of a 28-year-old male with COMS linked to a periapical lesion and supernumerary molars is presented. Treatment involved extracting specific teeth while preserving adjacent ones and using A-PRF for immediate OAC closure. A-PRF, enriched with growth factors, was pivotal in healing, showcasing enhanced tissue regeneration, pain reduction, and faster recovery. The findings suggest A-PRF as an effective adjunct in treating extensive OAC and COMS, proposing its inclusion in standard treatment protocols. This study underscores A-PRF’s potential in improving outcomes for patients with COMS and related complications.

## 1. Introduction

The maxillary sinus is a paired pneumatic space located in the body of the maxillary bone. It is formed already in fetal life. It develops and grows during childhood until all permanent teeth erupt. It performs important functions: cleansing, humidifying and heating the air, olfactory chamber, resonance cavity (gives the sound and strength of the voice), and is a space that reduces the weight of bone tissue. The sinus is lined with a mucous membrane covered with ciliated epithelium. This ensures proper drainage of the sinus. Also, it is the anatomical space where bone regeneration takes place after a sinus lift augmentation. The sinus mucosa (epithelium and vascular/perivascular layer) is believed to have osteogenic properties [1].

Chronic odontogenic maxillary sinusitis is an inflammatory process of the maxillary sinus, and it lasts longer than 12 weeks. It accounts for about 10–41% of types of sinusitis. The main odontogenic factors are: apical and marginal periodontitis, oro-antral communications (OACs) or fistulas (OAFs), and infection connected with foreign bodies (dental implants, biomaterials after bone-regeneration procedures) [2,3,4,5]. The progression of periapical lesions in maxillary lateral teeth may result in inflammatory changes in the mucosa of the maxillary sinus and, consequently, sinusitis [6]. *Streptococcus pneumoniae*, *Moraxella catarrhalis*, and *Haemophilus influenzae* are the most common pathogens implicated in chronic sinusitis [7]. Individuals afflicted with this condition often describe experiencing a blocked nasal passage, resulting in a reduced airflow and difficulty in nasal breathing, alongside a noticeable nasal discharge. Additionally, they report experiencing discomfort or pain, which can vary in intensity and location, and an impaired sense of smell, indicating potential olfactory disorders. Radiographic imaging has always played an imperative role in establishing the odontogenic etiology of chronic maxillary sinusitis and complement results of the clinical examination [6]. Cone beam computed tomography (CBCT) has become increasingly important tool in the diagnosis of odontogenic maxillary sinusitis [7]. The examination assesses the presence of thickened mucosa, discharge in the maxillary sinus, results of dental procedures (e.g., sinus lift), presence of OACs or OAFs, foreign bodies in the sinus (endodontic material, roots pressed into the sinus, etc.), and periapical lesions at the teeth located near the maxillary sinus. The management of sinusitis involves a multifaceted and comprehensive approach, primarily focusing on identifying and addressing the underlying cause of the condition. This may include the use of antibiotics for bacterial infections, corticosteroids to reduce inflammation, decongestants to alleviate nasal congestion, and in some cases surgical interventions to correct anatomical obstructions and facilitate drainage [2,3,4,5]. 

Platelet-rich preparations in medicine and dentistry were first described in the second half of the 20th century [8,9]. These are autogenous materials obtained from the patient’s peripheral blood containing leukocyte cytokines and platelets. Different types of preparations can be produced during the centrifugation process. The preparation of a given preparation depends on the time and speed of centrifugation as well as the weight of individual morphotic elements of blood and plasma. The first generation of platelet-rich preparations are platelet-rich plasma (PRP) and plasma-rich in growth factors (PRGF). The second generation of platelet-rich preparations are platelet-rich fibrin (PRF), concentrated growth factors (CGF), and autologous fibrin glue (AFG). The platelet-rich fibrin consists of: leukocyte- and platelet-rich fibrin (L-PRF), advanced platelet-rich fibrin (A-PRF), and injectable platelet-rich fibrin (I-PRF). In the process of centrifugation, platelets are activated to release growth factors. Growth factors stimulate the regeneration of soft and hard tissues [10,11,12,13,14,15]. 

Preparation of advanced platelet-rich fibrin is quick and easy. Before the procedure, blood should be collected from a peripheral vein. This is most often the cephalic or basilic vein. A-PRF is produced by centrifuging the patient’s blood in glass vacuum tubes without anticoagulants for 14 min at 1500 rpm. The centrifugation speed for A-PRF is lower than for PRP and the PRF net formation is a result of a natural process of a coagulation cascade. The essence of PRF formation is that fibrinogen is deposited in the upper part of the test tube before thrombin converts it into fibrin. PRF test tubes must not contain an anticoagulant. Thanks to this, the coagulation process begins when blood meets the glass test tube. However, contact with the silicone surface is necessary to activate the clot polymerization process [11,16,17,18,19]. A-PRF forms a hemostatic plug and slowly releases growth factors. It has a solid consistency and enables the release of growth factors for up to 4 weeks which stimulates wound healing. In the case of PRP, growth factors are released at the time of administration. A-PRF is autologous material which consist of fibrin matrix, rich in platelets and leukocytes, containing cytokines, stem cells, growth factors (platelet-derived growth factor (PDGF), transforming growth factor beta 1 (TGFβ1), insulin-like growth factor (IGF-1), vascular endothelial growth factor (VEGF), fibroblast growth factor (FGF) and epidermal growth factor (EGF)), and monocytes which release additional bone morphogenetic proteins (BMP-2 and BMP-7). This material is constituted a biodegradable scaffold, contributed to microvascularization development, and encouraged epithelial cells migration to its surface [10,12,13]. A-PRF stimulates angiogenesis by means of a release of growth factors [12,13,20]. 

The applications of A-PRF are diverse, encompassing several procedures in oral surgery like sinus floor elevation, ridge augmentation, socket preservation, bone defects, furcation defects, or in secondary alveolar bone grafting for cleft alveolus. Fibrin membranes could be better scaffolds for the proliferation of periosteal and osseous cells than collagen membranes in vitro. A-PRF can be applied to a surgical wound or dental alveolar as a clot, it can be compressed and used as a membrane or core or cut into fragments and mixed with bone substitute material [20,21,22,23,24]. 

The aim of this article was to evaluate the role of A-PRF in the treatment of odontogenic maxillary sinusitis and immediate closure of extensive oro-antral communication. 

## 2. Case Presentation

A 28-year-old patient was admitted to the Outpatient Clinic of Oral Surgery, University Dental Center of Medical University of Gdańsk, for consultation and treatment. The patient reported pain of maxilla on the left side, around the second molar—tooth 27 according to the FDI classification (fr. *Federation Dentaire Internationale*) and toothache.

**Figure 1:**
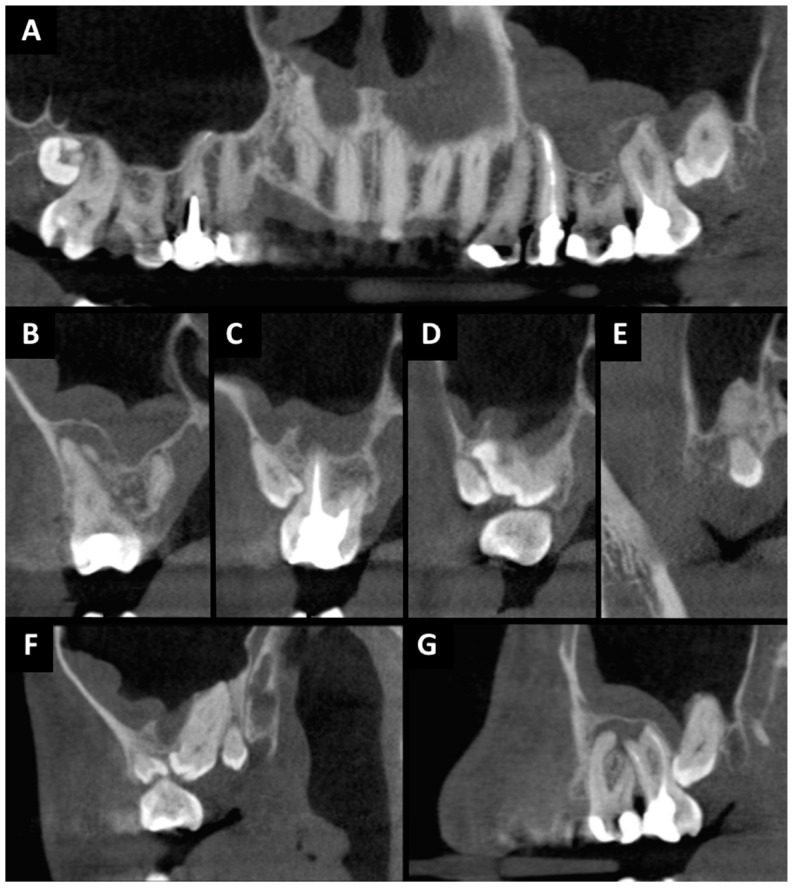
CBCT before surgery treatment: (**A**) pantomographic reconstruction: chronic odontogenic sinusitis on left side with odontogenic cyst related to tooth 27; (**B**) cross-sectional view: tooth 27 region; (**C**) cross-sectional view: tooth 27 and 29′ region; (**D**) cross-sectional view: tooth 27, 28 and 29′ region; (**E**) cross-sectional view: tooth 29″ region; (**F**) sagittal view: tooth 27, 28, 29′ and 29″ region; (**G**) sagittal view: tooth 26, 27 and 28 region.

The patient was generally healthy, without systemic diseases and allergies, non-smoker, and presenting with good oral hygiene. The extraoral, intraoral and CBCT examinations were performed. The extraoral examination revealed no abnormalities. The patient reported periodic nasal congestion and the appearance of rhinitis. In intraoral examination, tooth 27 showed an increased response to the vertical percussion test. The mucosa around the causative tooth was unchanged. The CBCT scan was then assessed using the CS 3D Imaging v3.5.18 Software (Carestream Health Inc., Trophy, Croissy-Beaubourg, France; Figure 1). The imaging conditions were 84 kV, 5 mA, voxel size of 0.2 mm, field of view (FOV) of 7 × 10 cm and CTDIvol 4.62 mGy. Due to the presence of third (tooth 28), fourth (tooth 29) and fifth (tooth 29’) molars during the development of the roots of teeth 26 (first molar) and 27, the supplementary teeth were an obstacle to the development of the tooth roots. As a result, the roots of teeth 26 and 27 were highly curved. This prevented proper endodontic treatment. Tooth 27 was root canal treated and had a periapical lesion that convexed into the maxillary sinus and chronic inflammation was visible. Buccal root has not been properly treated due to the large root curvature (90°). Root canal treatment was unsuccessful. Tooth 26 was healthy, with no pathological lesions. The roots of tooth 29 were ‘intertwined’ with the roots of tooth 27 and the patient was qualified for extraction of teeth 27 and 29 with the simultaneous preservation of teeth 26, 28 and 29’. Tooth 28 was left to replace tooth 27 in the dental arch.

The patient signed an informed written consent to the procedures and to the use of the data and photos for publication. A few days before the surgery, the patient rinsed his mouth with 0.2% chlorhexidine solution to reduce the number of microorganisms. Before the procedure, due to the risk of connecting the oral cavity with the maxillary sinus, the patient took a double dose of antibiotics (amoxicillin 0.875 g with clavulanic acid 0.125 g) one hour before the procedure.

**Figure 2:**
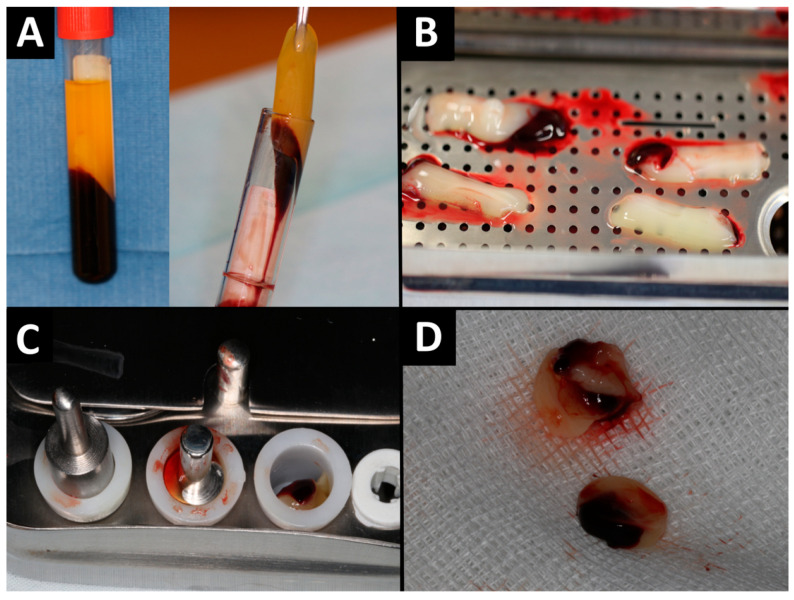
A-PRF preparation: (**A**) A-PRF on tubes; (**B**) A-PRF clots on PRF Box; (**C**) formation of A-PRF corks; (**D**) A-PRF corks.

The patient presented with the current blood count on the day of the procedure, which did not show any abnormalities. Before surgery, venous blood was collected from the patient (40 mL venous blood into four sterile, anticoagulant-free, glass-coated plastic tubes—10 mL each) in a separate office. Blood was taken from the cephalic vein. Then, tubes were immediately centrifuged by the centrifuge (All Centrifuge, Scilogex, LLC, Rocky Hill, CT, USA). Tubes must not be shaken or inverted before centrifugation. The time from blood collection to centrifugation cannot exceed 2 min. It is related to the lack of anticoagulant in the blood collection tube and the blood must be centrifuged before it will start coagulating. Centrifugation time was 14 min, and the speed was 1500 rpm. The above rules must be followed, otherwise a clot cannot be obtained. After centrifugation, 4 fibrin clots were achieved. A-PRF clots were removed from blood tubes, then was dissected by scissors from red blood cells base at the bottom, 2 millimeters below connection between layers. The connection area is rich in platelets, growth factors and leukocytes. After that A-PRF clots were put in special PRF Box (Quadrostom, Kraków, Poland). The spontaneous compression of A-PRF is very important. After positioning the clot in the PRF Box, allow the weight to act on its own. The material was formed into four corks (Figure 2). The A-PRF clot can be used up to 4 hours after centrifugation.

**Figure 3:**
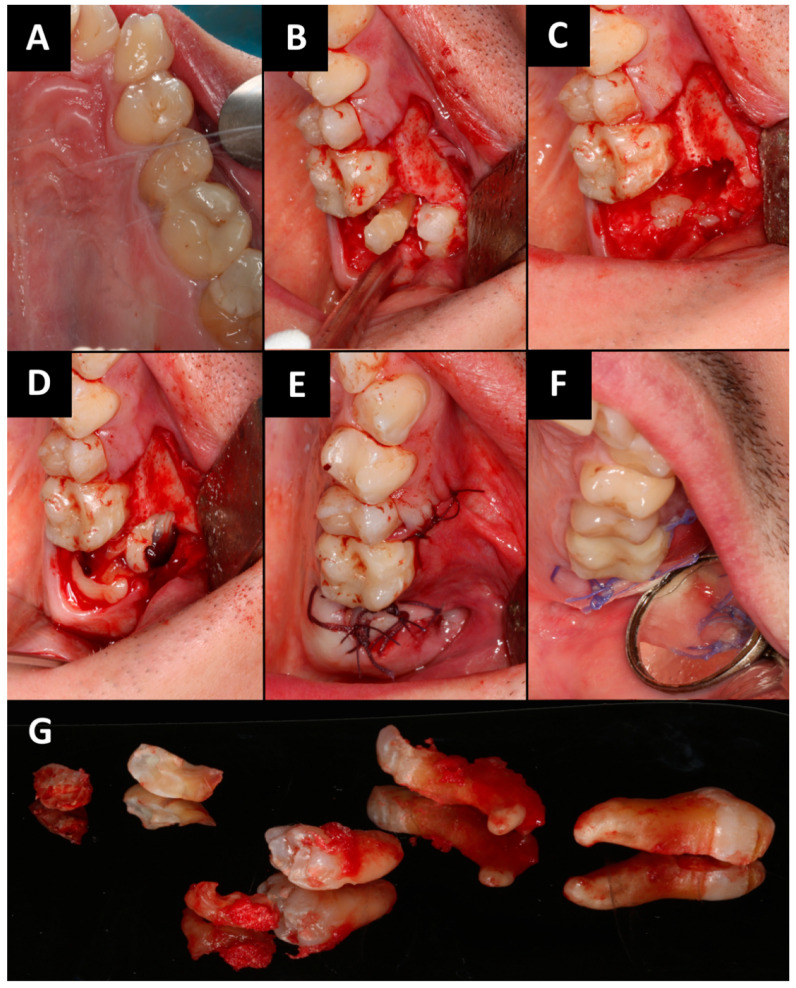
Intraoral photographs (**A**–**F**): (**A**) initial situation; (**B**) mucoperiosteal flap elevation, osteotomy and crown-root separation of tooth 27; (**C**) after minimally invasive removal of teeth 27 and 29; (**D**) A-PRF clots were placed in the wound; (**E**) the wound was sutured; (**F**) the wound healing after 14th day; (**G**) removed teeth 27 and 29.

**Figure 4:**
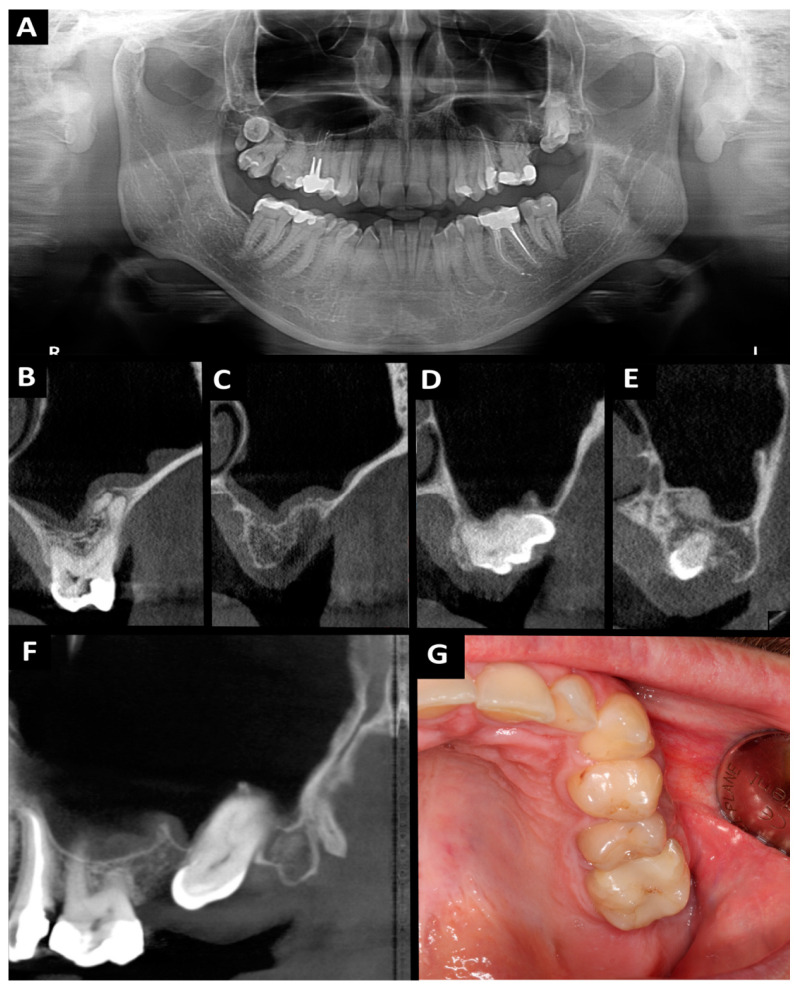
Orthopantomography (**A**) and CBCT (**B**–**G**) 10 months after the surgery: (**A**) orthopantomography; (**B**) cross-sectional view: tooth 26 region; (**C**) cross-sectional view: tooth 27 and 29′ region; (**D**) cross-sectional view: tooth 28 region; (**E**) cross-sectional view: tooth 29″ region; (**F**) sagittal view: tooth 26, 28 and 29″ region; (**G**) intraoral photograph.

The surgical procedure (Figure 3) was performed under local anesthesia which was given via injection. Posterior superior alveolar and palatal nerves were blocked using 2.4 mL 4% articaine hydrochloride containing 1:100,000 epinephrine (2 ampules; Citocartin 100, Molteni Dental s.r.l., Scandicci, Florence, Italy). Tooth 29′ was removed in a minimally invasive manner. Then trapezoidal incision was made with no. 15c scalpel blade and mucoperiosteal flap was raised. Tooth 27 was cut using a round bur mounted on an implanted surgical low-speed handpiece (W&H Dentalwerk Bürmoos GmbH, Bürmoos, Austria), at 1200 rpm under abundant irrigation (0.9% NaCl). After crown-root separation, the roots were removed minimally invasive using luxing periotom (Pol-Intech, Łódź, Poland). The cyst associated with the roots of tooth 27 was enucleated using bone curettes and forceps. Lesion was fixed in 10% formalin and commissioned for histopathological examination. The presence of an oro-antral communication was confirmed with mechanical test by an alveolar spoon and the Valsalva maneuver. A-PRF clots were placed in the wound. The oro-antral communication closure procedure was made. The wound was sutured tightly and without tension. Absorbed polyglycol sutures 4-0 and 3-0 were used. Hemostasis was achieved. A gauze pad was put on and the patient was instructed to bite on it and hold it for 20 min. For the prevention of postoperative edema, pain, and trismus, kinesio tapes (KT) were applied. A 5 cm wide plaster (cotton, acrylic adhesive, hypoallergenic) was used [25,26]. KT was divided into four parts, with one common end. The central KT was tightened by 15%. The KT was applied to clean skin and was applied from the clavicle (common end) to the zygomatic arch (free ends). The second KT scan was applied horizontally along the zygomatic arch. The patient was prescribed antibiotics (amoxicillin 0.875 g with clavulanic acid 0.125 g, every 12 h for 7 days), analgesics (nimesulide 0.1 g, twice daily in case of pain) and 0.1% xylometazoline (three times a day to each nasal vestibules for 5 days). In the peri-operative period, mouthwash with 0.2% chlorhexidine solution was recommended twice a day for 10 days. The patient had follow-up visit after 24 h and 14 days. Wound healing was normal, without pain. The sutures were removed on 14 day. The histopathological examination revealed: odontogenic cyst and fragments of the mucosa of the maxillary sinus in chronic inflammation. Ten months after the procedure, the patient came for a follow-up visit. The patient did not report any problems in the oral cavity and sinuses. The intraoral examination showed complete healing of the soft tissue. CBCT and photographic images were taken (Figure 4). Radiographic examination revealed a good bone healing at the site of surgery, there are no changes in the mucosal lining of the maxillary sinus floor, and it is not thickened.

## 3. Discussion

The frequency of oro-antral communication occurrences varies between 0.31% and 4%, with the highest likelihood noted during molar extractions, particularly the first molars, in the upper jaw. This is followed by premolars, and least frequently, canines. The main risk factors of OAC include: extensive alveolar recess of maxillary sinus, widely spaced teeth roots, the presence of periapical lesion and inflammation of the mucosa of the maxillary sinus, the force used during extraction on the upper jaw or another iatrogenic effects such as dental implant complications (e.g., after implant explantation), migration of dental implants and/or grafting materials into the maxillary sinus, osteonecrosis after radiotherapy and medication related osteonecrosis of the jaw, fractures in maxillofacial region (after fractures and after using mini-plates for osteosyntesis), and maxillofacial surgery procedures (placing mini-implants and mini-plates for orthodontic and orthognathic purposes) [7,27,28,29,30,31,32,33,34,35,36,37,38,39,40,41,42,43,44]. In our case, the development of OAC was favored by the presence of periapical lesion and maxillary sinus inflammation.

For managing oro-antral communication, employing a buccal or palatal flap for soft tissue closure remains the preferred method when primary closure via gingival suturing fails to effectively seal the orifice. Among these, the buccal flap technique, including the Rehrmann flap and the Wassmund-Borusiewicz method, is favored despite its potential to diminish the depth of the buccal sulcus. This preference is noted in comparison to the palatal flap approach, which, while effective, leads to the exposure of the donor site on the palate and necessitates secondary epithelialization for healing. The buccal flap procedure specifically entails the preparation of a trapezoidal mucoperiosteal flap. This process involves an incision through the periosteum and delineation of the gingival margin on the palatal side, a technique known as Borusiewicz’s correction, to ensure precise and effective coverage of the OAC. The flap requires careful mobilization to ensure that it is repositioned without any tension, which is critical for optimal healing and to minimize the risk of dehiscence. Following mobilization, the flap should be securely anchored in place using a horizontal mattress suture technique, providing robust, evenly distributed support across the wound. This initial suturing is crucial for maintaining the flap’s stability and alignment. Subsequently, individual interrupted sutures should be placed to further secure the flap and to fine-tune the adaptation of the tissue edges, ensuring a precise and snug fit that promotes effective healing. Nevertheless, many surgeons seem to prefer the palatal flap because of its excellent blood supply and the fact that the buccal sulcus remains intact. In contrast, a reduction of the buccal sulcus depth is currently becoming less of a problem with the possibility of implant-retained overdentures [28].

Within the realm of surgical repair, particularly in addressing OACs, the utilization of a pedicled flap integrated with a connective tissue graft (CTG) sourced from the premolar area of the palate stands out for its patient acceptability. This technique is favored due to the significantly reduced discomfort it causes compared to the transplantation of a full-thickness flap from the same region. Conversely, the free gingival graft (FGG) technique, which entails the extraction of both gingiva and connective tissue from the palate, emerges as an effective strategy for sealing smaller OACs. However, its application is limited in cases of more extensive defects, where the risk of FGG necrosis becomes a concern [42]. Furthermore, the buccal fat pad (BFP)—also referred to as Bichat’s fat pad—presents a novel treatment modality by employing adipose tissue from the cheek. This method can be employed either as a standalone procedure or in conjunction with the Rehrmann flap. A distinctive advantage of utilizing the BFP by itself is its non-interference with the oral vestibule, which facilitates subsequent denture placement without complication. Although this approach is generally well-received by patients, it is important to note that it can only be applied once per treatment side, limiting its repeatability [45,46]. Other less popular methods include: autogenous distant flaps (tongue flap, auricular cartilage, septal cartilage, temporalis muscle flap), autogenous bone grafts (the bone can be collected extraorally (crest of the iliac plate, fibula, ribs, tibia, skull bones) or intraorally (retromolar triangle area, area between the mandibular foramina, maxillary protuberance, external oblique line in mandible, edentulous section of the jaws (e.g., under the pontic of the bridge), or surgical area)), allogenous (fibrin glue, dura), xenograft (collagen, gelatin film, xenogenous bone grafts and membranes), synthetic materials and metals (gold, aluminum, tantalum, polymethylmethacrylate, hydroxyapatite, root analogue, titanium dental implant), other methods (tooth autotransplantation, interseptal alveolotomy, prolamin gel, splint, prosthetic dentures, biostimulation with laser light) [42,47,48,49,50].

Another promising technique may be the use of growth factors. Especially in connections up to 5 mm, they can become a simple and quick therapeutic method. Platelet-rich preparations stimulate healing and angiogenesis, reduce the postoperative reaction, pain, and inflammatory complications. The first reports on this subject are already being described. So far, only a few studies using PRP and PRF to close oro-antral communications/fistulas have been described. These are mainly case reports. There are no studies discussing the problem of treatment of odontogenic maxillary sinusitis and growth factor applications. The most frequently utilized treatment for OAC and OAF involves a combination of platelet-rich fibrin and a tension-free buccal flap, or alternatively, the sole use of PRF. The results of treatment with growth factors were very good and all patients had good healing in follow-up [30,31,32,33,34,35,36,37,38,39,40,48,49,50,51,52,53,54,55]. In our patient, we also achieved very good treatment results and, additionally, healing of inflammatory lesions in the maxillary sinus in the case of odontogenic chronic sinusitis.

A-PRF may be a healing aid in patients with extensive OAC. Platelet concentrates provide a supernatural number of cells including 90% platelets and 50% leukocytes when compared to their concentration in whole blood [38]. The blood centrifugation within a specific glass-based tube leads to the activation of the physiological coagulation cascade [39]. A-PRF is a platelet concentrate consist of an autologous bioscaffold of a dense three-dimensional fibrin meshwork with naturally integrated growth factors which are released from the scaffold through the natural maturation and reorganization of the clot over a sustained period to promote healing of hard and soft tissues [40]. It is characterized by the gradual release of growth factors, up to one month. In addition, it contains higher concentrations of growth factors than other platelet-rich preparations. That is of key importance for the regeneration of bones and soft tissues [36]. A-PRF was developed with the low-speed centrifugation concept (LSCC). It means that lower G-forces are using to obtain higher growth factor release when compared to standard PRF and PRP. By reducing the centrifuge speed, leukocyte infiltration into the red blood cell fraction is also minimized. The leukocytes in PRF help in elimination of necrotic debris thereby preventing infection at the defect site. They also help in recruitment of undifferentiated mesenchymal cells native to the site being regenerated and totipotent cells from the cambial layer of the periosteum by upregulation of cytokines and chemokines. This reduction of G-force also resulted in increased levels of cytokines which help in cellular recruitment and monocytes that help in osteoblastic differentiation [38].

After analyzing the available literature and the assessment of our patient, the role of platelet growth factors in the treatment of oro-antral communication and fistulas may be important. Early and accurate diagnosis is crucial in managing the condition effectively. However, this article extends the discourse by incorporating recent advancements in diagnostic and treatment technologies, offering fresh insights into potential therapeutic ways. The described clinical case shows the role of growth factors in the treatment of chronic odontogenic maxillary sinusitis. Currently, there are insufficient studies on a large group of patients with OACs and OAFs treated with growth factors. Typically, these investigations are conducted on a limited cohort of patients and employ a comprehensive approach that incorporates not only growth factors but also various supplementary surgical techniques. Future research directions should focus on large cohort studies using only clots and membranes of platelet-rich fibrin, platelet-rich plasma or concentrated growth factors, as well as clinical and radiological assessment of the maxillary sinuses before and after treatment (e.g., potassium permanganate discoloration during sinus rinsing or CBCT examination and assessment of the thickness of the maxillary sinus mucosa or the presence of effusion). The case report described in this article shows new directions of research and their degree of advancement on the way to implementation to the human clinic. 

## 4. Conclusions

Oro-antral communication is a frequently encountered complication during the extraction of teeth from the lateral sections of the maxilla. This article marks a significant milestone as it introduces the innovative application of advanced platelet-rich fibrin for effectively sealing OAC and addressing chronic maxillary sinusitis. The utilization of A-PRF in this context represents a groundbreaking approach, offering substantial promise as a therapeutic adjunct in the comprehensive management of patients afflicted with extensive OAC and concomitant inflammatory conditions in the maxillary sinus. Such advancements underscore the potential of A-PRF to revolutionize treatment paradigms, paving the way for enhanced healing outcomes in complex dental and maxillofacial interventions.

## Data Availability

The data presented in this study are available on request from the corresponding author. The data are not publicly available due to privacy restrictions.

## Abbreviations

AFG	autologous fibrin glue
A-PRF	advanced platelet rich fibrin
BAF	buccal advanced flap
BFP	buccal fat pad
CBCT	cone beam computed tomography
CGF	concentrated growth factors
FGF	fibroblast growth factor
GBR	guided bone regeneration
I-PRF	injectable platelet-rich fibrin
KT	kinesio tapes
L-PRF	leukocyte-platelet-rich fibrin
OAC	oro-antral communication
OAF	oro-antral fistula
OPG	orthopantomography
PD-EGF	platelet-derived epidermal growth factor
PDGF	platelet-derived growth factor
PRF	platelet-rich fibrin
PRP	platelet-rich plasma
TGF	transforming growth factor
TGFβ-1	tumor necrosis factor β-1
VEGF	vascular-endothelial growth factor
4PM	first premolar
5PM	second premolar
6M	first molar
7M	second molar
8M	wisdom tooth
